# Educating public health physicians for the future: a current perspective from Aotearoa New Zealand

**DOI:** 10.1186/1743-8462-6-7

**Published:** 2009-04-09

**Authors:** Chris Bullen, Pat Neuwelt

**Affiliations:** 1 School of Population Health, The University of Auckland, Auckland, New Zealand

## Abstract

Persisting, and in some cases widening, inequalities in health within and between countries present significant challenges to the focus and practice of contemporary public health, and by association, to public health education. As public health physicians and academic educators of medically- and non-medically trained public health practitioners, we call for a radical re-think of current approaches to public health medicine education and training in order to address these challenges. The public health physicians of the future, we argue, require not merely technical knowledge and skills but also a set of values that underpin a commitment to ethical principles, social equity, human rights, compassionate action, advocacy and leadership. Furthermore, while they will need to have their action firmly grounded in local realities they should think, if not speak and act, from an informed awareness of global issues. Drawing from our experience in Aotearoa New Zealand, as well as with marginalised communities overseas, we proffer our suggestions for the process and content of public health physician education and training for the future, with the intention of stimulating debate.

## Introduction

Persisting, and in some cases widening, inequalities in health within and between countries present significant challenges to the focus and practice of contemporary public health, and by association, to public health education. As public health physicians and academic educators of medically- and non-medically trained public health practitioners, we call for a radical re-think of current approaches to public health medicine education and training in order to address these challenges. Drawing from our experience in Aotearoa New Zealand, as well as with marginalised communities overseas, we proffer our suggestions for the process and content of public health physician education and training for the future. This paper does not address in detail the technical skills required of public health physicians. Such skill sets have been addressed through the development of professional competencies, such as those developed in New Zealand for the New Zealand College of Public Health Medicine [1].

## Discussion

Public health is by its very nature an eclectic enterprise. It is a collective movement that draws on people from across many disciplines and sectors working in concert to achieve its goals of protecting and improving the health of populations. In this paper we focus our attention on public health physicians, the medical actors in public health, whilst acknowledging that they are only one group of actors. Public health physicians are medical practitioners from a wide range of medical specialities with specific training in the principles and practice of public health. They are in a unique position to contribute to public health through their understanding of, and familiarity with, medical practice. This familiarity allows them to straddle the worlds of clinical and public health. As physicians they are often selected for leadership roles in the public health sector and accorded status and privilege by society that, we argue, can be a potent tool for effecting positive change on a stage much wider than just that of the health sector.

One of the major challenges facing educators of future public health physicians is that the biomedical world view which doctors bring into public health training potentially constrains their contribution to the public health movement. In the 1960s–90s, public health had a strong focus on the identification of individual-level risk factors through the use of epidemiologic techniques. Accordingly, public health action emphasised changing individual behaviour. Theories of causation were largely individually-oriented, and the now widely-accepted notion of the social determinants of health [2] was not prominent in academic discourse [3]. The biomedical world view was theoretically consistent with the "concepts and framework of what might be called epidemiologic theory" ([3], p. 887). In contrast, the 'new' public health as articulated by Baum and others incorporates a strong social determinants perspective with social equity as its core [4].

We contend that a commitment to competent and ethical practice requires that the underpinning values of public health be fostered in the education and training of public health physicians. What are these values and what are the implications for education and training? Should public health physicians be concerned about human *need *or human *rights *or both? Core values for the future practice of public health medicine, we believe, include ethical practice, social equity, human rights, and compassionate action. It can be argued that these represent a particular political perspective, that of collectivism rather than individualism. Such a perspective underpins what has been called 'social or community medicine'. We will consider each of these in turn, beginning with a discussion of ethical medical practice in public health.

### Ethical practice

Physicians need education and training in ethical frameworks for public health decision-making in order to be effective public health practitioners. As health leaders, public health physicians are required to have the skills necessary to make difficult judgements in the face of ambiguity. Many decisions involve balancing the public health needs of populations with the needs of individuals. While the major ethical principle underpinning clinical medicine is individual autonomy or rights, public health emphasises utilitarianism or the greatest good for the greatest number. In the words of Kass [5],

The most important asset that public health can have is the public's trust that work is being done on its own behalf. In such a context, public health professionals can and must advocate what they believe, on balance, are the ethically best approaches for furthering social justice and the public's health (p. 1782).

Callahan and Jennings ([6], p. 175) argue that ethics teaching must be added to teaching the science of public health.

It also must be made on the basis of a conception of the qualities and abilities that a public health professional should possess if he or she is to be truly educated toward public service: sound judgement, ability to recognize and analyze ethical issues, tolerance for ambiguity, and capacity for a moral imagination with which seemingly isolated issues or events can be placed in a broader context of human experience and value.

Childress et al [7] have presented a set of 'general moral considerations' which, they argue, are relevant to ethical public health practice:

• producing benefits;

• avoiding, preventing, and removing harms;

• producing the maximal balance of benefits over harms and other costs (often called utility);

• distributing benefits and burdens fairly (distributive justice) and ensuring public participation, including the participation of affected parties (procedural justice);

• respecting autonomous choices and actions, including liberty of action;

• protecting privacy and confidentiality;

• keeping promises and commitments;

• disclosing information as well as speaking honestly and truthfully (often grouped under transparency); and

• building and maintaining trust (p. 172).

The challenge for students and practitioners comes in the implementation of these moral considerations or ethical principles/values, particularly when they sit in conflict with one another. Although the field of 'public health ethics' is relatively new and underdeveloped relative to biomedical ethics, conceptual frameworks for ethical public health practice are currently being developed by a number of scholars (see, for example, [8] and [5]). Further, some tertiary institutions are incorporating them into public health education and training. Given the current challenges facing public health in the global arena, the time is nigh for public health physicians to be trained in more than technical competence, but also to make sound ethical judgements. Public health ethics needs further development and implementation.

### Social equity

As part of a commitment to ethical practice, public health physicians of the future will need a commitment to social equity. As mentioned previously, the 'new public health' places equity at the very core of public health concerns. In his book, Pathologies of Power, Farmer [9] states, "Equity is the central challenge for the future of medicine and public health" (p. 20). He argues that a public health ethics framework must incorporate a global perspective; "In discussion of medical ethics, global health equity has become the elephant in the room that no one mentions" (p. 208). In short, a commitment to distributive justice requires that public health physicians are educated and trained to consider power and privilege not only on the local stage, but the global. We agree. Educating public health practitioners to understand and act upon the major determinants of ill-health must include a critique of the inequitable distribution of health determinants that lead to health and other inequalities, locally and globally.

Inequity has been defined as "systematic inequality in health (or in its social determinants) between more and less advantaged social groups, in other words, a health inequality that is unjust or unfair" ([10], p. 255). Baum ([4], p. xiv) sees social equity as an approach which attempts to break down barriers between professional groups and between professionals and lay people. Further, she posits that social equity rejects the ideology of individualism upon which medical practice is based and recognises that social and political structures constrain people's access to not only the determinants of good health but also to health care services.

As a high proportion of physicians have come from privileged backgrounds, it is important that public health physician training programmes include exposure to social or structural analysis. This challenges the 'downstream-ism' of clinical medicine. In most nations, physicians have been conferred significant privileges. In many western nations, physician education is publicly subsidised, and doctors are assured of high incomes and social standing throughout their careers. In light of this, physicians have, we argue, a moral responsibility to use both their individual and collective privilege and power to improve equity in society.

### Human rights

An understanding of privilege in society, and a commitment to addressing the needs of those most disadvantaged, must also incorporate a human rights perspective. There has been in increasing recognition of the value of a human rights approach to the understanding and practice of public health issues over the past decade. Mann [11] argued over a decade ago, "Progress in the new public health, based on awareness that societal factors determine, more than anything else, who lives and who dies, of what and when, requires further development of human rights analysis and methods of action" (p. 1941). A human rights analysis in the health setting involves recognising violations of the right to health and to the social determinants of health. In Mann's [11] words,

This approach would consider a whole human being made vulnerable to a wide variety of pathogens and unhealthy conditions as a result of how the person is treated by society – expressed and articulated in the language of human rights and dignity (p. 1940).

Mann argued, further, that a human rights approach would ensure that public health put due emphasis on prevention, despite the demands of health emergencies, illness and injury. Further, such prevention efforts would be undertaken in an ethical manner, using a variety of approaches, selected and designed locally by the people concerned.

In New Zealand, the rights-based approach has come to the fore in public health in recent years with regard to the health experience of Māori, the indigenous people of this land. While an equitable public health approach would prioritise the needs of Māori simply on the basis of their need for better health, a human rights (and indigenous rights) approach would prioritise Māori on the basis of the right of Māori to enjoy at least as good health as non-Māori New Zealanders[12].

To apply a human rights analysis to our public health learning and practice, we must be both self-reflexive and engaged in collective learning and action. Mann [11] argues that the true task of public health practitioners is to call the larger societal status quo into question. He continues:

Perhaps paradoxically...this constant challenge to the societal status quo first requires that we re-examine the status quo within ourselves. It is difficult to challenge the "givens" of an economic system, of political power, or of religious or cultural traditions. We can do so only if we are anchored by something within ourselves – and if we are linked, connected, and nourished by others (p. 1942).

The ability to be self-reflexive, and to work as part of supported public health collective action, is what allows public health physicians to engage in action for positive social change. The addition of human rights analysis to public health education and training will enhance the ability of public health physicians to act effectively, thoughtfully and compassionately.

### Compassionate action and advocacy

Rudolf Virchow's historic assertion that 'physicians are the natural attorneys of the poor' is a call for physicians to speak up, to bear witness, on behalf of those unable to do so themselves [9]. It is our assertion that public health physicians in particular should be trained in such advocacy, for as Baum [13] asserts, the complex global public health challenges of the century require an *increase *in advocacy for equity. Public health physicians also have an important role in supporting other physicians to be informed advocates in their areas of expertise (what we would call 'secondary advocacy'). It has been recognised that those employed by government agencies may be unable to engage widely in advocacy, but that those self-employed or working for more independent agencies have a responsibility to speak out [14].

To be an effective advocate in the public arena requires a personal and professional commitment to using specialist knowledge and insights to improve the health of populations. Further, advocacy brings the personal into the professional and vice versa. An advocate is someone who can empathise or have compassion for the people on whose behalf he/she is advocating. Thus, it is critical that public health physicians-in-training have the opportunity to learn the realities of 'ordinary people' and their struggles, or as Farmer puts it to make 'common cause with the poor'. As public health physicians progress into senior roles in the health sector they can all too easily lose touch. In order for public health physicians to stay grounded in the realities of life for the majority, the training programme should facilitate engagement between physicians and local communities. We now turn to this point in greater detail.

### Local and global

We argue that public health physicians must be not only in touch with local communities, particularly those with high needs, but also be globally aware. Globalisation is arguably the defining word of the 21st century but is it good for health, and if so, whose health[15]? Feachem's [16] assertion that 'globalisation is good for health, mostly', begs the question: how will the health of the poor fare in the new global order?

As with the nuclear armaments debate, doctors have a valid role to play in the debate about globalisation and health [17]. But are they equipped and informed to take part? We believe that public health education should equip physicians to not only act locally but also to think, if not speak and act, from an informed awareness of global issues. They must act and advocate at the local level in communities, and nationally; they must also do so internationally, while at the same time being aware of the implications and impacts of global processes and forces on local health. In the words of Farmer [9],

It's difficult enough to think globally and act locally, but perhaps what we are really called to do ... is to think locally *and *globally and to act in response to both levels of analysis (p.159).

To do so credibly and competently, it is hard to go past first hand experience. Therefore, training opportunities that involve working with communities of high need, locally and overseas, should be supported and indeed, actively promoted. In such settings, where the ability to engage effectively with a wide range of individuals and groups is critical, public health medicine trainees learn how to communicate public health concepts in accessible language. Public health physicians are uniquely placed among doctors to build bridges – between civil society and the health sector, between communities and policymakers. But they will only be truly effective if they can translate both ways: turning complex concepts into more meaningful language is one thing, but listening to and understanding lay perspectives is perhaps the harder task.

Being 'culturally competent' – having the attitudes, skills and knowledge needed to function effectively and respectfully when working with and treating people of different cultural backgrounds – is key [18]. Such competence only comes from spending time in communities with people. Petersen and Lupton [19] argue the need for health workers to better understand lay perspectives in health. We would go further to assert that lay and indigenous people should be included as key public health educators.

## Implications for education and training

If public health physicians of the future are to have the understanding and skills to work as 'attorneys of the poor' then, as we have noted, there are significant implications for their education (learning to think) and training (learning to do). We draw this distinction between education and training to make the point that what physicians require most of all as they embark on public health practice is additional education and the development of 'critical faculties'. The selection of candidates, the content and setting of their education and training, and the process of training are all important to consider. We address these in turn.

### The Selection of Candidates

Public health physician education and training begins with the selection of appropriate candidates. The underlying philosophy of the public health medicine training organisation is a significant determinant of the future public health medicine workforce. We argue that training programmes should give priority to candidates who display not only intellectual ability but also qualities that are important for effective public health practice: a commitment to social equity and leadership ability within teams. Without these qualities, they will struggle to engage effectively in public health education, training, and subsequent practice.

A commitment to social equity can be evident in clinicians' stories of their medical practice. In our experience, those clinicians who have worked with high needs populations have less difficulty making the shift, as they have encountered the sense of frustration in providing clinical services as 'the ambulance at the bottom of the cliff'. Without such experience, clinicians entering public health medicine may find it difficult to make the shift to considering structural factors in society that enhance or limit people's ability to make healthy choices.

Public health physicians must increasingly play a key leadership role not only in the health sector, but in the wider public sector. The best candidates will therefore be individuals who demonstrate the ability to work effectively within teams, and who bring leadership qualities to those teams.

### The Content

The public health physician programme in New Zealand includes both a university component (completion of the Masters in Public Health degree) and employment-based supervised work experience, both under the rubric of 'public health medicine training' [20]. The university component provides a unique opportunity for education. First, physicians learn public health principles and values alongside colleagues from a wide range of non-medical backgrounds. Any assumptions about disciplinary superiority are soon challenged in this environment. While there is still a focus on individual achievement, they learn that multidisciplinary team work is an essential dimension of effective public health practice. Second, they are encouraged to think critically, read widely and articulate clearly their emerging understanding of health from a range of newly acquired perspectives. If the public health physicians of the future are to adopt a social equity approach, they must be taught how to analyse societies in terms of power and privilege. They need to be challenged to consider the broader social determinants of health, such as poverty, housing, education and racism.

Next, several years are spent in supervised practice in a range of workplaces, such as in academic units, health policy, planning and funding organisations and regional public health services. In these settings there is a greater focus on training than education. Nevertheless, this time is vital to the socialisation of public health physicians. Perhaps the most important element of this component is the opportunity to work alongside other public health practitioners, including public health physician supervisors and, in the process, acquire values as well as the key skills relevant to public health practice.

It has been asserted that public health education and training must ensure that students are "socialised in the values of public health" ([14], p. 219). Clearly, having supervisors committed to fostering public health values and taking action on the basis of public health ethical principles is critical to the success of this part of the socialisation process. An additional opportunity that has great potential for influence over this time is the required engagement of trainees with mentors; senior public health physicians who provide broad guidance around career goals and pathways and who ideally role model many of the values.

### The Setting

As we have argued above, the training of public health physicians for the future must include tertiary postgraduate education as well as supervised time in a 'public health medicine apprenticeship'. We have presented a case for a greater local experience with communities. We further contend that opportunities to experience public health practice in a developing country should also be encouraged and indeed, actively facilitated. Exposure to a wide variety of trainers/teachers including social scientists, community agency staff, community leaders, local government employees and so on is important. Models of shared training between public health and primary care practitioners, for example, should be explored. More radically we argue the need to make the whole health workforce a public health workforce, with public health physicians playing a key leadership role.

## Conclusion

What, then, of public health physician education and training? Training programmes must aim for not merely technical competency, but also the ability to make sound judgements in situations of ambiguity for the good of the public health. A multi-disciplinary setting is essential to gaining a breadth of exposure to the diversity of the public health workforce, public health thinking and debates. They must learn alongside students of other backgrounds, be taught by people from different disciplinary backgrounds, and be 'socialised in the values of public health'.

While a firm grounding in the principles of the investigation and management of public risk, communicable and non-communicable disease control, and health systems is a given, we maintain that technical competency is only one element in shaping the public health physicians of the future. We have outlined a case for a public health education that occurs through the lens of social equity so that public health medicine practitioners become well-informed 'activists' and opinion leaders in the health sector and in society, where they can model and advocate a commitment to improving social equity through health. We have argued that in order for this to happen, those entering public health medicine need to be socialised in public health values and approaches, developing skills in structural analysis, community engagement, public health ethical analysis and in advocacy, particularly around the social determinants of health, for as Virchow asserted

"...if medicine is really to accomplish its great task it must intervene in political and social life. It must point out the hindrances that impede the normal social functioning of vital processes and effect their removal."

(Virchow 1848 cited in ([9], p. 323)

## Competing interests

The authors declare that they have no competing interests.

## Authors' contributions

CB and PN conceptualised the paper and shared equally in its drafting.
